# Supplementary material for: The Link between Abdominal Obesity Indices and the Progression of Liver Fibrosis: Insights from a Population-Based Study
Source: Nutrients. 2024 May 23;16(11):1586. doi: 10.3390/nu16111586 (PMC11174397; doi:10.3390/nu16111586)
Supplement: Supplementary file 1 [file nutrients-16-01586-s001.zip › nutrients-3004076-supplementary.pdf]

Supplementary Figure S1. Changes in MASLD (a) and liver fibrosis (b) according to changes in BMI between baseline and follow-up.

(a)

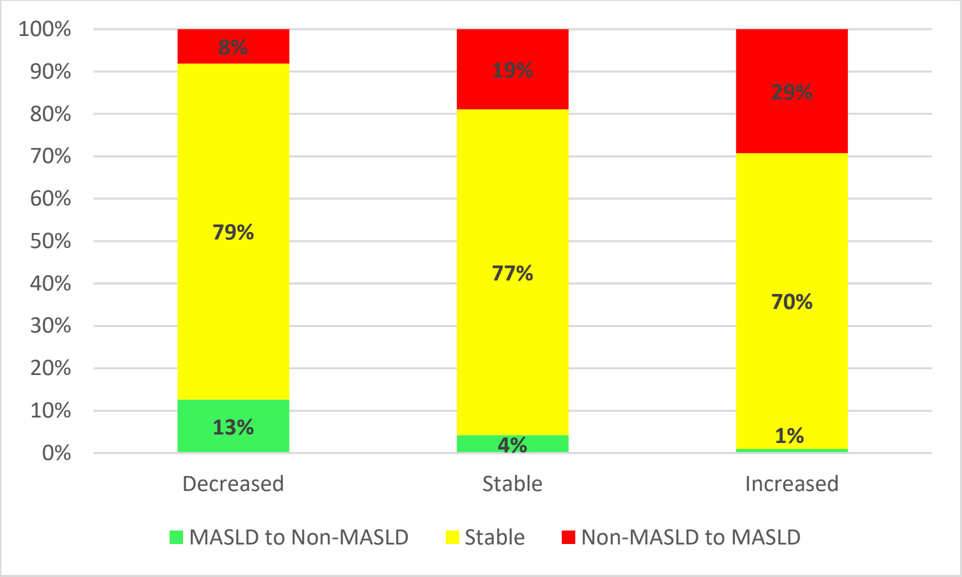

(b)

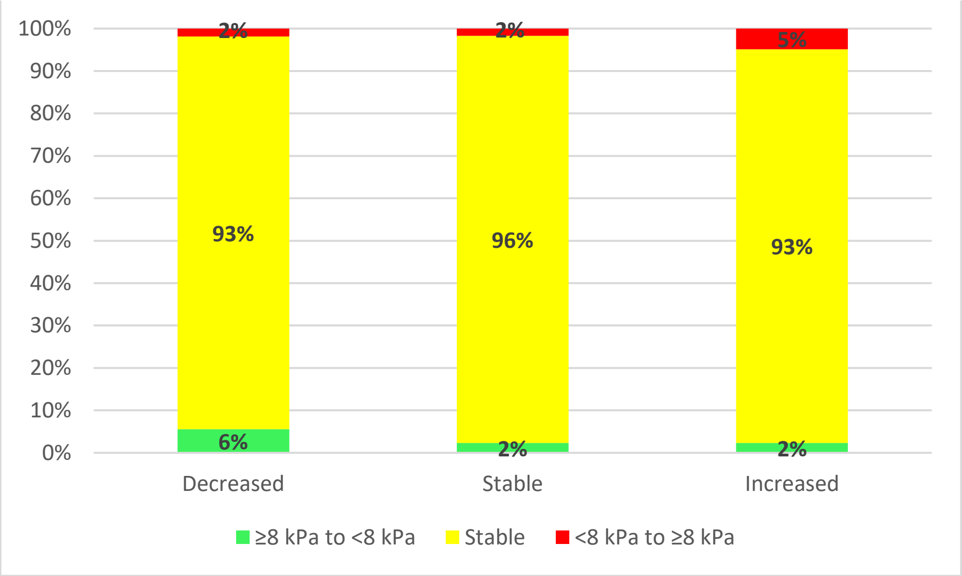

Figure legend: Participants were categorized into three groups based on BMI changes over time: decrease (reduction in BMI ≥ 1 kg/m^2), stable (reduction or increment in BMI < 1 kg/m^2) and increase (increment in BMI ≥ 1 kg/m^2). (a) Changes in MASLD according to changes in BMI ($p < 0.001$). (b) Changes in fibrosis according to changes in BMI ($p = 0.001$).

Supplementary Table S1. Changes in longitudinal LSM by stage at follow up.

**Baseline**	**Follow-Up**			
	<8 kPa		≥8 kPa	
	n	%	n	%
<8 kPa	1297	97%	38	3%
≥8 kPa	43	63%	25	37%

[Change must be of ≥1 kPa]
